# Supplementary material for: Examining the Feasibility of an Application-Based Patient-Reported Outcome Monitoring for Breast Cancer Patients: A Pretest for the PRO B Study
Source: Int J Environ Res Public Health. 2022 Jul 7;19(14):8284. doi: 10.3390/ijerph19148284 (PMC9324292; doi:10.3390/ijerph19148284)
Supplement: Supplementary file 1 [file ijerph-19-08284-s001.zip › ijerph-1662838-supplementary.pdf]

Additional File

Examining the feasibility of an application-based patient-reported outcome monitoring in breast cancer patients: a pretest for the PRO B study

Anna Maria Hage[1]*, Pimrapat Gebert[2,3]*, Friedrich Kühn[1], Therese Pross[1], Ulrike Grittner[2,3]*and Maria Margarete Karsten[1]*

[1] Charité – Universitätsmedizin Berlin, Department of Gynecology with Breast Center, Berlin, Germany

[2] Berlin Institute of Health at Charité – Universitätsmedizin Berlin, Charitéplatz 1, 10117 Berlin, Germany

[3] Charité – Universitätsmedizin Berlin, Institute of Biometry and Clinical Epidemiology, Berlin, Germany

*These authors contributed equally

Correspondence: Dr. med. Maria Margarete Karsten
Charité – Universitätsmedizin Berlin
Department of Gynecology with Breast Center
Charitéplatz 1
10117 Berlin, Germany
Tel.: +49 (0)30 450 564 255
Fax: +49 30 450 527 937
Email: pro-b-projekt@charite.de

Table S1. Pretest evaluation questionnaire.

Part A: Overall Opinion about PRO B Questions

	**Not at all**	**A little**	**Moderate**	**Much**	**Very Much**
1) How satisfied were you with the survey overall?					
2) Were the questions understandable to you?					
3) Were the questions relevant to you?					
4) Were you able to concentrate the entire time during the survey?					
5) Was the duration for completing the survey appropriate?					

	**No**	**Yes**	**If yes, please provide more detail.**
6) Are there any questions that are repetitive or very similar?			
7) Are important aspects of cancer symptoms missing from the survey?			
8) What did you like about the survey?			
9) What did you dislike about the survey?			
10) Do you have any suggestions for improvement?			

Part B: Overall Opinion on Using the Application

	1	2	3	4	5	6	7
	**Totally disagree**	→					**Totally agree**
**Ease of use and satisfaction**							
The app was easy to use							
It was easy for me to learn to use the app							
I like the interface of the app							
The information in the app was well organized, so I could easily find the information I needed							
I feel comfortable using this app in social settings							
The amount of time involved in using this app has been fitting for me							
I would use this app again							
This app offers great opportunity to improve care for breast cancer patients							
Overall, I am satisfied with this app							
**System information arrangement**							
Whenever I made a mistake using the app, I could correct it easily and quickly							
The navigation was simple and clearly structured							
**Technical implementation**							
Did you have problems using the app?	☐ Yes		☐ No (End the questionnaire)				
In what area did you have a problem with?	☐ Installation<br>☐ While using the app<br>☐ Other …………………………………………						
Could you describe the problem?	***Free text***						
How did you solve the problem?	☐ With help from someone in the the family/friend/colleague<br>☐ By contacting the study team or the developer of the app<br>☐ Searching on the internet<br>☐ By myself<br>☐ Other …………………………………………						
Do you have any suggestions for improving the app?	***Free text***						

Table S2. A semi-structured telephone interview.

The following questions were used as a guide for the telephone interview with the patient.

Questions about the PRO B questionnaire

- How did you feel about answering these questions related to your health in the app once a week?
- How long did it take to answer all the questions?
- What do you think if the patients in the upcoming PRO B study have to answer these questions about their health status once a week for a year?
- Do you think the questions cover all relevant aspects of the breast cancer patient?
- Do you think that the answers to the questions will be helpful for the physicians to know the patient's health status?
- Did you notice a question that you could not answer or you were uncomfortable to answer?
- Have you ever forgotten to answer the questions in the app?
- Did you have any problems with the app? What is the problem? How did you solve it? How long did it take you to solve the problem? What is your general opinion about the app? Does it has all the functions? Is something missing in the app and you would like to have it?
- Would you continue to use this app in the future?
- Would you recommend other patients to use this app?
- Do you think that patients who are not on the intervention arm (with alarm) will answer the questions every three months? If not, how could you suggest us to motivate and support these patients to stay in the study?
